# The Association between Long Working Hours and Self-Rated Health

**DOI:** 10.1186/2052-4374-26-2

**Published:** 2014-01-20

**Authors:** Jun-Taek Song, Goeun Lee, Jongho Kwon, Jung-Woo Park, Hyunrim Choi, Sinye Lim

**Affiliations:** 1 Department of Occupational and Environmental Medicine, Kyung Hee University Hospital, Seoul 130-872, Republic of Korea

**Keywords:** Long working hours, Weekly working hours, Self-rated health

## Abstract

**Objectives:**

This study was conducted to determine the number of hours worked per week by full-time wage workers by using the data of the Korean Labor and Income Panel Study (KLIPS), which represents the domestic urban area household, and to determine the association between weekly working hours and the level of self-rated health.

**Methods:**

We used data from the 11th KLIPS conducted in 2008. The subjects of this study were 3,699 full-time wage workers between the ages of 25 and 64 years. The association between weekly working hours and self-rated health was analyzed considering socio-demographic characteristics, work environment, and health-related behaviors.

**Results:**

Among the workers, 29.7% worked less than 40 hours per week; 39.7%, more than 40 to 52 hours; 19.7%, more than 52 to 60 hours; and 10.9%, more than 60 hours per week. After controlling for socio-demographic variables, work environment-related variables, and health-related behavior variables, the odds ratio (OR) for poor self-rated health for the group working more than 40 hours and up to 52 hours was calculated to be 1.06 (95% confidence interval (CI), 0.89-1.27) when the group working less than 40 hours per week was considered the reference. The OR for the group working more than 60 hours was 1.42 (95% CI, 1.10-1.83) and that for the group working more than 52 hours and up to 60 hours was 1.07 (95% CI, 0.86-1.33). After stratification by gender and tenure, the OR of the female workers group and that of the group with a tenure of more than 1 year were found to be significantly higher than those of the other groups.

**Conclusions:**

This study showed that workers working more than 60 hours per week have a significantly higher risk of poor self-rated health than workers working less than 40 hours per week. This effect was more obvious for the female workers group and the group with a tenure of more than 1 year. In the future, longitudinal studies may be needed to determine the association between long working hours and various health effects in Korean workers.

## Introduction

The relationship between working hours and health is an important issue in the field of occupational research. Many previous studies have shown that long working hours have negative impacts on workers’ health. It has been reported that workers working long hours have a higher risk of myocardial infarction [1,2], non-insulin dependent diabetes (NIDD) [3], and retiring due to disabilities [4]. Working long hours not only affects a worker’s health but also has a negative effect on the worker’s family [5,6]. Further, it has been reported to cause an increase in the number of accidents and errors in the workplace [7,8]. Caruso et al. presented a conceptual framework for the study of long working hours and stated that long working hours affect sleep and recovery, heighten job demands, increase the time of being exposed to dangerous factors in the workplace, and cause physiological changes, having adverse effects on the workers’ health [5].

The International Labour Organisation (ILO) founded in 1919 presented “Controls on Working Hours as an Urgent Improvement Subject” in its constitution at the time of its founding [9]. Then, in November 1996, the European Community implemented regulations on working hours, including the rights of a laborer to refuse to work more than 48 hours a week [10]. According to the Fifth European Working Conditions Survey published in 2012, as a result of such efforts, the number of laborers working more than 48 hours per week has decreased steadily among wage workers in the European countries [11]. In Korea, the number of working hours in a week cannot exceed 40 hours excluding the break time according to the Labor Standards Act, while overtime work hours are regulated to not exceed 12 hours per week [12]. However, despite such regulations, a recent annual report of the statistics of the Organization for Economic Co-operation and Development (OECD) revealed Korea’s average annual working hours to be 2,193 hours in 2010, ranking number one among OECD countries [13].

The self-rated health level is used widely as an indicator of the health condition of an individual, as it reflects the present state of an illness as well as the general health condition of an individual [14,15]. In a research conducted in the US, it was reported that the odds ratio (OR) of workers working 70 hours or more per week who rated their general health condition and answered that their condition was poor or fair as compared to workers working 35–40 hours per week with the same response was 1.94 (95% confidence interval (CI), 1.07-3.51) [16]. Despite the fact that Korea has the longest annual average working hours among the OECD countries, not many studies have been conducted on the relationship between working hours and their impact on workers’ health. Some studies on working hours and mental health have been conducted, including studies on long working hours conducted using domestic data showing that workers working long hours have a higher risk of suicidal ideation [17] or show depressive symptomatology [18,19]. However, there have been few studies conducted on the effects of long working hours on general health or self-rated health. Therefore, this study has been conducted using the Korean Labor and Income Panel Study (KLIPS) data to analyze the working hours of full-time wage workers to determine the association between long working hours and self-rated health.

## Study population and methods

KLIPS was funded by Ministry of Employment and Labor and conducted with the approval of the Statistics Korea (approval number: 33601). KLIPS was a publicly opened dataset and did not contain any personal information, therefore ethical approval for this study was waived.

### Study population

The research data of the 11th KLIPS conducted in 2008 have been used in this study. KLIPS represents 10% of the sample survey population from the 1995 census on total population and housing, on the basis of a study on 5,000 households living in non-rural areas and panel sample members (all members of households living in the 5,000 households) representing household members. Further, KLIPS is a longitudinal study that includes follow-up surveys on panel sample members’ economic activities, labor movement, income, expenditures, education, job training, and social activities, surveyed once a year starting from 1998 [20].

**Figure 1:**
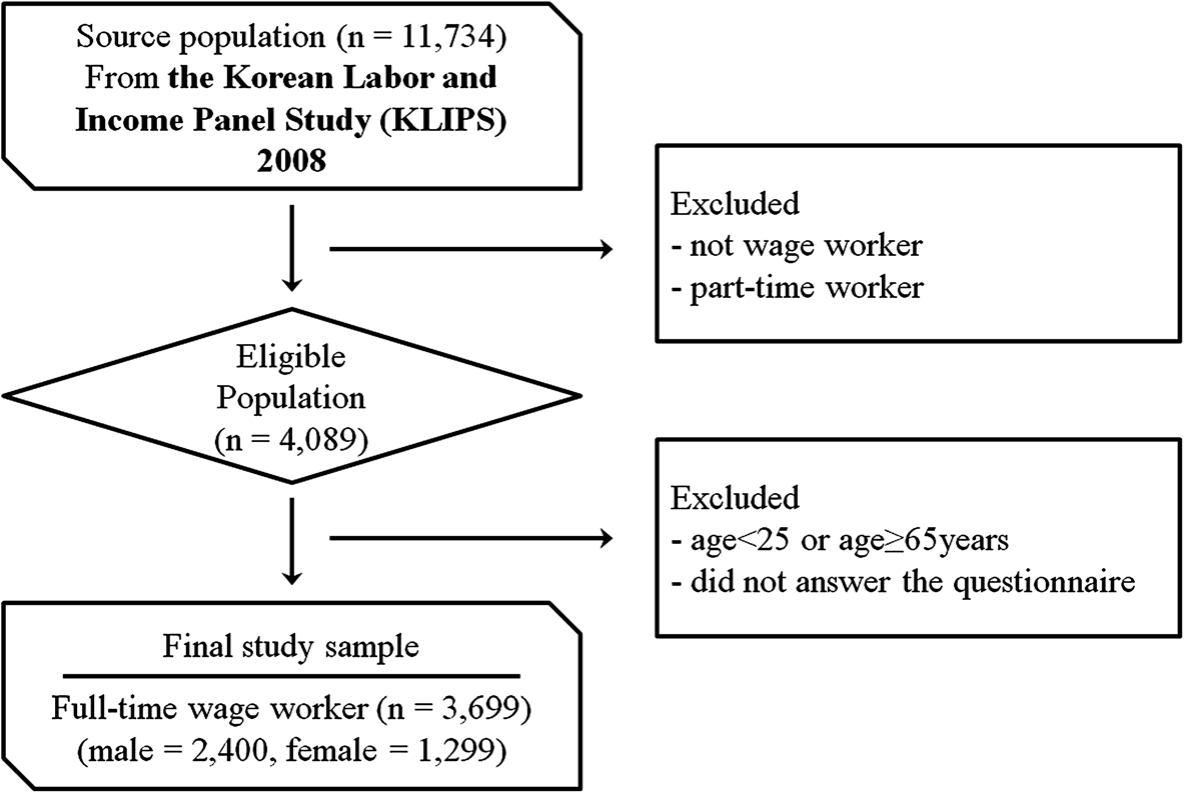
Flowchart of study population.

Among the 11,734 study subjects of the 11th KLIPS conducted in 2008, the subjects of this study were 4,089 people who reported that they were full-time wage workers. The study subjects have been restricted to those between the age of 25 years or above and less than 65 years, considering the employment age of university graduates and the retirement age of workers, so that, after excluding the missing data, the study participants finally included 3,699 people (Figure 1).

## Materials and methods

### Working hours

Under Korea’s Labor Standards Act, working hours are limited as follows: The first clause of Article 50 states that “Working hours per week shall not exceed 40 hours, excluding break times”, while the first clause of Article 53 states that “If the parties concerned reach agreement, the working hours stipulated in Article 50 may be extended by up to 12 hours per week”, allowing a total number of working hours of up to 52 hours under the law [12]. Moreover, in a study on the approval criteria of cardiovascular diseases caused by overwork, if the number of working hours per week right before an occurrence of a cardiovascular disease exceeds 60 hours, then the case is defined as short-term overwork [21].

In this study, the study subjects have been categorized into a less than 40 hours group, more than 40 hours and up to 52 hours group, more than 52 hours and up to 60 hours group, and more than 60 hours group, on the basis of the Labor Standards Act and overwork standards. For cases with a fixed number of regular working hours, the number of working hours per week has been estimated by adding the answers of “What are your current regular working hours per week excluding meal times?” and “In your current job, how many hours do you work overtime on average in a week?” For cases without a fixed number of regular working hours, the answer to the question “In your current job, how many hours do you work on average in a week?” has been used.

### Outcome variable

The outcome variable of self-rated health has been surveyed on a scale of five levels including “very good”, “good”, “fair”, “poor”, and “very poor”, on the basis of evaluation conducted by workers on their own at the time of the study. Among the five levels, “very good” and “good” were defined as the “healthy group”, while the remaining levels were defined as the “unhealthy group”.

### Measurement of covariates

Other individual variables including the participants’ socio-demographic characteristics, occupational characteristics, and items related to health-related behavior characteristics have been reconstructed by category. Gender, age, marital status, educational status, and monthly household income were chosen as the variables of the socio-demographic characteristics. The marital status variable had the values of “single”, “married with a spouse”, and others (“separated”, “divorced”, and “widow/widower”). The educational status was categorized into a below junior high school group, high school group, and above college group after answering the question “What is your highest level of education? Or are you currently studying?” The monthly household income variable has six portions including earned income, financial income, real estate income, social insurance income, transfer income, and other income with the monthly income calculated by adding all portions of income divided by the square root of the number of family members.

Occupation, employment type, shift work status, and tenure have been chosen as the occupational variables. In the case of the occupation variable, “legislative officials”, “senior officials and managers”, “specialists”, “engineers and associate experts”, and “office workers” have been categorized as non-manual workers, while the remaining occupations including “service workers” and “sales workers” have been categorized as manual workers. The employment type variable has been categorized into regular workers and irregular workers on the basis of the answers chosen by the workers, while the shift work variable was categorized into the shift work group for those who answered that they worked in shifts and the daytime work group for those who answered that they did not work in shifts. The tenure variable has been categorized into the below one year group, the more than one year up to 10 years group, and the more than 10 years group.

Smoking, alcohol consumption, and exercise have been chosen as the variables for health-related behavior characteristics. In the case of the smoking variable, subjects who answered “currently smoking” were categorized as the smoking group, while those who answered “smoked in the past but quit smoking” and “never smoked before” were categorized as the non-smoking group. In the case of the alcohol consumption variable, subjects who answered “currently drinking” were categorized as the alcohol-consuming group, while those who answered “drank in the past but quit drinking,” and “never drank before” were categorized as the non-alcohol-consuming group. In the case of the exercise variable, subjects who answered “yes, I exercise” were categorized as the exercise group, while those who answered “hardly exercise” were categorized as the non-exercise group.

### Statistical analysis

Frequency analysis and the chi-squared test were conducted in order to understand the relationship between the participants’ working hours and each individual variable as well as the relationship between self-rated health and each individual variable. It has been predicted that gender and cumulative exposure time will be found to be important contributors in the relationship between the number of working hours and self-rated health. Female workers were expected to have different characteristics from male workers on the basis of a previous study showing that female workers earn lower wages or have less job control than male workers [22] and the cumulative exposure time is believed to have a great impact on self-rated health because the number of working hours closely affects the workers’ lifestyle. On the basis of this fact, the following three types of models were formed and logistic regression analyses were carried out after controlling the relevant variables:

**Model 1**: Logistic regression analysis model of the study participants after controlling for the socio-demographic characteristics, occupational characteristics and health-related behavior characteristics.

**Model 2**: Logistic regression analysis model of the study participants after controlling for the relevant variables after gender stratification.

**Model 3**: Logistic regression analysis model of the study participants after controlling for the relevant variables after tenure stratification.

SPSS statistics 20.0 (IBM Corp., Armonk, NY, USA) has been used for the statistical analysis, with a significance level of less than 5%.

## Results

### General characteristics of the study participants

Among the 3,699 study participants, there were 2,400 (64.9%) male participants and 1,299 (35.1%) female participants, with 29.7% of them in the group working less than 40 hours per week, 39.7% in the group working more than 40 hours and up to 52 hours per week, 19.7% in the group working more than 52 hours and up to 60 hours per week, and 10.9% in the group working more than 60 hours per week (Table 1). The average working hours per week for all the study participants was 48.8 hours, with longer working hours per week for male workers, manual workers, regular workers, and shift workers than for female workers, non-manual workers, irregular workers, and daytime workers.

**Table 1:** Weekly working hours by study subjects’ characteristics

**Variables**		**Total**	**Weekly working hours**			
			**≤40**	**40 < × ≤ 52**	**52 < × ≤ 60**	**>60**
		**N Mean ± SD**^**†**^	%^*^Mean ± SD^**†**^	%^*^Mean ± SD^**†**^	%^*^Mean ± SD^**†**^	%^*^Mean ± SD^**†**^
Total		3,699	29.7	39.7	19.7	10.9
		48.8 ± 12.5	36.8 ± 6.8	47.0 ± 2.8	57.1 ± 2.8	73.5 ± 11.0
Gender	Male	2,400	28.5	39.7	20.7	11.1
		49.2 ± 12.7	36.7 ± 6.8	47.0 ± 2.8	57.1 ± 2.8	74.1 ± 11.9
	Female	1,299	31.8	39.9	17.8	10.5
		48.2 ± 12.0	36.9 ± 6.9	46.9 ± 2.8	57.3 ± 2.8	72.3 ± 8.7
Age	25-34	1,248	30.0	41.2	18.7	10.1
		48.7 ± 12.0	37.5 ± 6.3	47.0 ± 2.8	57.2 ± 2.8	73.4 ± 11.6
	35-44	1,139	28.9	40.5	19.9	10.7
		49.1 ± 12.8	36.7 ± 7.1	47.1 ± 2.7	57.0 ± 2.8	74.9 ± 11.7
	45-54	897	29.9	37.1	20.8	12.2
		49.0 ± 12.7	36.3 ± 6.9	47.0 ± 2.8	57.1 ± 2.8	72.4 ± 10.3
	55-64	415	30.4	38.8	19.5	11.3
		48.4 ± 12.6	36.0 ± 7.2	46.4 ± 2.7	57.3 ± 2.8	72.9 ± 8.3
Occupation	Non-manual	1,802	31.8	43.2	16.1	8.9
		47.8 ± 11.7	37.2 ± 6.6	46.7 ± 2.8	57.1 ± 2.8	73.6 ± 10.4
	Manual	1,897	27.6	36.5	23.0	12.9
		49.9 ± 13.1	36.3 ± 7.0	47.3 ± 2.7	57.2 ± 2.8	73.5 ± 11.4
Employment type	Regular worker	2,767	28.4	40.9	19.6	11.1
		49.1 ± 12.4	37.2 ± 6.6	46.9 ± 2.8	57.0 ± 2.8	73.9 ± 11.2
	Irregular worker	932	33.4	36.2	19.9	10.5
		48.0 ± 12.8	35.9 ± 7.3	47.0 ± 2.7	57.4 ± 2.8	72.5 ± 10.1
Shift work	No	3,330	30.3	40.0	19.3	10.4
		48.5 ± 12.4	36.7 ± 6.9	46.9 ± 2.8	57.1 ± 2.8	73.3 ± 11.0
	Yes	369	24.1	37.4	22.8	15.7
		51.6 ± 13.1	38.0 ± 5.1	47.2 ± 2.8	57.1 ± 2.7	74.8 ± 10.6
Tenure	≤1	988	28.9	41.0	18.8	11.3
(years)		49.0 ± 12.7	36.6 ± 6.7	47.1 ± 2.8	57.4 ± 2.8	74.0 ± 10.7
	1 < × ≤ 10	2,013	29.6	38.3	20.4	11.7
		49.1 ± 12.8	36.8 ± 7.1	47.0 ± 2.7	57.1 ± 2.7	73.5 ± 11.6
	>10	698	30.8	42.2	18.8	8.2
		47.8 ± 11.1	37.1 ± 6.2	46.8 ± 3.0	56.8 ± 2.8	72.6 ± 8.6

*: row percentage, †SD: standard deviation.

In the cases of both male and female workers, there was no significant difference found between each working hour group with respect to age distribution and marital status (Tables 2 and 3). With respect to educational status, the group having completed high school or beyond was the largest percentage in the group working more than 40 hours and up to 52 hours per week for female workers. In contrast, in the case of male workers, the group with an educational status of below junior high school showed the largest percentage in the group working less than 40 hours per week (p < 0.001) (Tables 2 and 3). With respect to the monthly household income, both male and female workers showed the highest income level in the group working less than 40 hours per week; in the case of male workers, the monthly household income tended to drop significantly as the number of working hours increased (p < 0.001), but the female group showed no statistically significant difference.

**Table 2:** General characteristics of the male subjects

**Variables**		**Total**	**Weekly working hours**				**p-value**
			**≤40**	**40 < × ≤ 52**	**52 < × ≤ 60**	**>60**	
		n(%^*^)	**%**^**†**^	**%**^**†**^	**%**^**†**^	**%**^**†**^	
Age	Mean ± SD^‡^	40.0 ± 10.0	41.5 ± 10.2	40.8 ± 9.9	40.8 ± 9.9	41.1 ± 9.9	0.479^§^
(years)	25-34	759(31.6)	27.3	40.7	20.5	11.5	0.811^∥^
	35-44	786(32.8)	27.0	40.8	21.4	10.8	
	45-54	566(23.6)	31.5	36.7	20.5	11.3	
	55-64	289(12.0)	30.1	39.5	19.7	10.7	
Marital status	Never married	520(21.7)	28.3	41.9	20.0	9.8	0.196^∥^
	Married	1,766(73.6)	28.4	39.7	20.6	11.3	
	Others	114(4.7)	31.6	28.1	25.4	14.9	
Educational status	≤Junior high school	304(12.7)	34.2	29.6	22.7	13.5	<0.001^∥^
	High school	819(34.1)	25.8	37.6	23.8	12.8	
	≥College	1,277(53.2)	28.9	43.4	18.2	9.5	
Household income (KRW/month)¶	Mean ± SD^‡^	207.7 ± 260.5	224.6 ± 302.9	214.4 ± 273.7	190.1 ± 209.4	173.6 ± 155.4	<0.001^**^
Occupation	Non-manual	1,097(45.7)	29.4	44.0	17.6	9.0	<0.001^∥^
	Manual	1,303(54.3)	27.8	36.0	23.3	12.9	
Employment type	Regular worker	1,879(78.3)	27.0	41.0	20.6	11.4	0.008^∥^
	Irregular worker	521(21.7)	34.0	34.7	21.3	10.0	
Shift work	No	2,110(87.9)	29.3	39.8	20.4	10.5	0.011^∥^
	Yes	290(12.1)	22.8	38.6	22.8	15.8	
Tenure	Mean ± SD^‡^	7.06 ± 7.58	7.46 ± 7.77	7.40 ± 7.99	6.42 ± 6.96	6.00 ± 6.48	0.122^**^
(years)	≤1	567(23.6)	27.3	40.4	21.2	11.1	0.015^∥^
	1 < × ≤ 10	1,288(53.7)	28.2	37.7	21.4	12.7	
	>10	545(22.7)	30.5	43.5	18.7	7.3	
Smoking	No	1,012(42.2)	30.1	41.7	19.0	9.2	0.008^∥^
	Yes	1,388(57.8)	27.3	38.2	22.0	12.5	
Alcohol consumption	No	370(15.4)	27.3	44.1	18.6	10.0	0.291^∥^
	Yes	2,030(84.6)	28.7	38.9	21.1	11.3	
Exercise	No	1,721(71.7)	27.6	37.6	22.3	12.5	<0.001^∥^
	Yes	679(28.3)	30.8	44.9	16.6	7.7	

*: column percentage, †: row percentage, ‡SD: standard deviation, §: tested by ANOVA, ∥: tested by chi-squared test,

¶:KRW = 10,000 Korean won (approximately US$10), **: tested by Kruskal-Wallis test.

**Table 3:** General characteristics of the female subjects

**Variables**		**Total**	**Weekly working hours**				**p-value**
			**≤40**	**40 < × ≤ 52**	**52 < × ≤ 60**	**>60**	
		n(%^*^)	**%**^**†**^	**%**^**†**^	**%**^**†**^	**%**^**†**^	
Age	Mean ± SD^‡^	39.8 ± 10.2	39.0 ± 10.1	39.5 ± 10.3	41.0 ± 10.0	41.3 ± 10.4	0.022^§^
(years)	25-34	489(37.6)	34.2	42.1	15.7	8.0	0.090^∥^
	35-44	353(27.2)	33.1	39.7	16.7	10.5	
	45-54	331(25.5)	27.2	37.8	21.4	13.6	
	55-64	126(9.7)	31.0	37.3	19.0	12.7	
Marital status	Never married	303(23.3)	34.0	39.9	16.2	9.9	0.252^∥^
	Married	858(66.1)	32.5	39.3	17.5	10.7	
	Others	138(10.6)	22.4	43.5	23.2	10.9	
Educational status	≤Junior high school	281(21.6)	27.1	34.5	24.9	13.5	<0.001^∥^
	High school	433(33.3)	27.5	42.5	19.4	10.6	
	≥College	585(45.1)	37.3	40.5	13.2	9.0	
Household income (KRW/month)^¶^	Mean ± SD^‡^	228.9 ± 333.6	241.7 ± 329.7	231.1 ± 362.8	209.2 ± 260.7	214.6 ± 340.9	0.640^§^
Occupation	Non-manual	705(54.3)	35.6	41.8	13.9	8.7	<0.001^∥^
	Manual	594(45.7)	27.3	37.5	22.4	12.8	
Employment type	Regular worker	888(68.4)	31.4	40.8	17.6	10.2	0.803^∥^
	Irregular worker	411(31.6)	32.6	38.0	18.2	11.2	
Shift work	No	1,220(93.9)	32.0	40.3	17.5	10.2	0.249^∥^
	Yes	79(6.1)	29.1	32.9	22.8	15.2	
Tenure	Mean ± SD^‡^	4.67 ± 5.57	4.71 ± 5.25	4.65 ± 5.82	4.62 ± 5.44	4.75 ± 5.78	0.995^§^
(years)	≤1	421(32.4)	30.9	41.8	15.7	11.6	0.775^∥^
	1 < × ≤ 10	725(55.8)	32.3	39.2	18.7	9.8	
	>10	153(11.8)	32.0	37.9	19.0	11.1	
Smoking	No	1,281(98.6)	31.7	40.1	17.7	10.5	0.463^∥^
	Yes	18(1.4)	38.9	22.2	22.2	16.7	
Alcohol consumption	No	631(48.6)	31.1	41.2	16.6	11.1	0.573^∥^
	Yes	668(51.4)	32.5	38.6	18.9	10.0	
Exercise	No	1,083(83.4)	31.1	40.5	17.3	11.1	0.223^∥^
	Yes	216(16.6)	35.2	36.6	20.3	7.9	

*: column percentage, †: row percentage, ‡SD: standard deviation, §: tested by ANOVA, ∥: tested by chi-squared test, ¶:KRW = 10,000 Korean won (approximately US$10).

With respect to the occupational characteristics of both male and female study participants, manual workers were more common than non-manual workers in the group working more than 52 hours per week (p < 0.001). With respect to the employment type, irregular male workers formed a higher proportion of the number of employees working less than 40 hours per week and a lower proportion of those working more than 40 hours and up to 52 hours per week than regular male workers (p = 0.008), while in the case of female workers, no significant difference between the groups by employment type was observed. Both male and female shift workers formed a higher proportion of the number of workers working more than 52 hours per week and a lower proportion of workers working less than 52 hours per week than daytime workers, although the difference was significant for just the males (p = 0.011). In the case of male workers, the percentage of participants working more than 52 hours per week was considerably lower for the group who had worked for more than 10 years than for the group who had worked for less than 10 years (p = 0.015). In contrast, in the case of female workers, the percentage of those working more than 52 hours per week was higher in the group who had worked for more than 10 years, even though the difference was statistically non-significant. With respect to the health-related behavior characteristic variables of the study participants, the smoking group, alcohol-consuming group, and non-exercising group formed a higher proportion of the number of male and female workers working more than 52 hours per week (Tables 2 and 3).

### The relationship between relevant variables and prevalence of poor self-rated health

In the case of male workers, the prevalence of poor self-rated health with respect to the number of working hours per week was 27.9% for the group working less than 40 hours per week, 26.2% for the group working more than 40 hours and up to 52 hours per week, 27.8% for the group working more than 52 hours and up to 60 hours per week, and 31.5% for the group working more than 60 hours per week, showing a U-shaped form with no statistically significant differences (Table 4). In the case of female workers, the prevalence of poor self-rated health was 32.7% for the group working less than 40 hours per week, 36.7% for the group working more than 40 hours and up to 52 hours per week, 40.3% for the group working more than 52 hours and up to 60 hours per week, and 51.8% for the group working more than 60 hours per week, showing a significantly higher rate in the unhealthy group for subjects working more hours per week with a dose–response pattern (p < 0.001). The self-rated health level with respect to the socio-demographic characteristics showed a significantly higher rate in the unhealthy group for older people and participants with lower educational status in the cases of both male and female workers (p < 0.001). With respect to the marital status variables, the people in the divorced, separated, or widow/widower group showed a higher unhealthy rate (p < 0.001). The self-rated health level with respect to occupational characteristics showed that manual workers had a higher unhealthy rate than non-manual workers in the cases of both male and female workers. With respect to the employment type, irregular workers had a higher unhealthy rate than regular workers (p < 0.001). In the cases of both male and female workers, there was no significant difference in the self-rated health level with respect to shift work. The unhealthy rate with respect to the number of years of tenure showed no significant difference in the case of male workers, but in the case of female workers, the group that had been working for less than one year showed a significantly higher unhealthy rate (p = 0.005). The self-rated health level with respect to the health-related behavior characteristics for male workers showed that the smoking group had a higher unhealthy rate but with no significant difference, while the non-alcohol-consuming group (p = 0.017) and the non-exercise group (p < 0.001) showed a significantly higher unhealthy rate. In the case of female workers, the smoking group and the non-exercise group showed a higher unhealthy rate, although statistically insignificant, while the non-alcohol-consuming group showed a significantly higher unhealthy rate (p = 0.019).

**Table 4:** Prevalence of poor self-rated health according to socio-demographic characteristics

**Variables**		**Total**		**Male**		**Female**	
		**Prevalence of poor self-rated health**	**p-value**^**†**^	**Prevalence of poor self-rated health**	**p-value**^**†**^	**Prevalence of poor self-rated health**	**p-value**^**†**^
		%^*^		%^*^		%^*^	
Weekly working hours	≤40	29.7	0.007	27.9	0.387	32.7	<0.001
	40 < × ≤ 52	29.9		26.2		36.7	
	52 < × ≤ 60	31.7		27.8		40.3	
	>60	38.4		31.5		51.8	
Age	25-34	22.2	<0.001	20.8	<0.001	24.3	<0.001
(years)	35-44	26.4		23.0		34.0	
	45-54	39.9		34.1		49.8	
	55-64	51.8		45.0		67.5	
Marital status	Never married	21.5	<0.001	19.8	<0.001	24.4	<0.001
	Married	31.7		28.3		38.8	
	Others	56.0		51.8		59.4	
Educational status	≤Junior	53.5	<0.001	46.7	<0.001	60.9	<0.001
	high school						
	High school	31.2		28.8		35.8	
	≥College	24.0		22.2		27.9	
Occupation	Non-manual	24.5	<0.001	22.7	<0.001	27.4	<0.001
	Manual	37.4		31.7		49.8	
Employment type	Regular worker	27.1	<0.001	24.5	<0.001	32.4	<0.001
	Irregular worker	43.1		38.6		48.9	
Shift work	No	31.1	0.796	28.6	0.673	37.3	0.307
	Yes	31.7		27.4		43.0	
Tenure	≤1	34.2	0.015	27.0	0.346	43.9	0.005
(years)	1 < × ≤ 10	30.8		28.7		34.5	
	>10	27.7		25.5		35.3	
Smoking	No	32.4	0.031	26.1	0.161	37.4	0.114
	Yes	29.0		28.7		55.6	
Alcohol consumption	No	37.9	<0.001	32.7	0.017	40.9	0.019
	Yes	28.6		26.7		34.6	
Exercise	No	37.4	<0.001	29.5	<0.001	37.9	0.722
	Yes	24.5		22.8		36.6	

*: row percentage.

†: tested by chi-squared test.

### The relationship between weekly working hours and self-rated health level

The odds ratio (OR) of the group with poor self-rated health level with respect to the weekly working hours was analyzed with the group working less than 40 hours used as a reference (Table 5). Model 1, after controlling for the variables of the study participants’ socio-demographic characteristics, occupational characteristics, and health-related behavior characteristics, showed an OR of 1.06 (95% CI, 0.89-1.27) for the group working more than 40 hours and up to 52 hours, 1.07 (95% CI, 0.86-1.33) for the group working more than 52 hours and up to 60 hours, and 1.42 (95% CI, 1.10-1.83) for the group working more than 60 hours.

**Table 5:** Odds ratios (OR) and 95% confidence intervals (CI) for poor self-rated health

		**Weekly working hours**	**Number of workers**		**OR (95% CI)**
			**Good health**	**Poor health**	
Model 1^*^		≤40	771	326	1.00
		40 < × ≤ 52	1,031	439	1.06 (0.89-1.27)
		52 < × ≤ 60	497	231	1.07 (0.86-1.33)
		>60	249	155	1.42 (1.10-1.83)
Model 2^†^	Male	≤40	493	191	1.00
		40 < × ≤ 52	703	249	1.00 (0.79-1.26)
		52 < × ≤ 60	359	138	0.98 (0.75-1.28)
		>60	183	84	1.14 (0.83-1.57)
	Female	≤40	278	135	1.00
		40 > x ≤ 52	328	190	1.17 (0.88-1.57)
		52 > x ≤ 60	138	93	1.22 (0.85-1.74)
		>60	66	71	1.97 (1.30-2.99)
Model 3^‡^	Tenure	≤40	180	105	1.00
	≤1 year	40 < × ≤ 52	277	128	0.88 (0.63-1.24)
		52 < × ≤ 60	122	64	0.93 (0.62-1.42)
		>60	71	41	0.95 (0.59-1.55)
	Tenure	≤40	430	167	1.00
	1 <**×** ≤ 10 years	40 < × ≤ 52	544	226	1.14 (0.89-1.46)
		52 < × ≤ 60	279	132	1.15 (0.86-1.55)
		>60	140	95	1.75 (1.25-2.45)
	Tenure	≤40	161	54	1.00
	>10 years	40 < × ≤ 52	210	85	1.27 (0.84-1.93)
		52 < × ≤ 60	96	35	1.05 (0.62-1.76)
		>60	38	19	1.37 (0.71-2.65)

*: Model adjusted for gender, age, marital status, income, educational status, occupation, employment type, tenure, shift work, smoking, alcohol consumption, and exercise.

†: Model 1 after stratification by gender.

‡: Model 1 after stratification by tenure.

Model 2 was constructed after gender stratification and then controlled for the relevant variables. In Model 2, the female workers with a poor self-rated health level showed an OR of 1.17 (95% CI, 0.88-1.57) for the group working more than 40 hours and up to 52 hours, 1.22 (95% CI, 0.85-1.74) for the group working more than 52 hours and up to 60 hours, and 1.97 (95% CI, 1.30-2.99) for the group working more than 60 hours, showing a higher OR of poor self-rated health level than the results of Model 1. However, in the case of male workers, the OR of 1.14 (95% CI, 0.83-1.57) was higher than the reference group only in the group working more than 60 hours.

Model 3 was constructed after tenure stratification and then controlled for the relevant variables. The OR of poor self-rated health level for workers with more than one year up to 10 years of tenure was 1.14 (95% CI, 0.89-1.46) for the group working more than 40 hours and up to 52 hours, 1.15 (95% CI, 0.86-1.55) for the group working more than 52 hours and up to 60 hours, and 1.75 (95% CI, 1.25-2.45) for the group working more than 60 hours, showing a higher OR of poor self-rated health level than the results of Model 1. However, the OR of workers with less than one year of tenure with a poor self-rated health level decreased and showed a U-shaped pattern. Further, in the case of workers with more than 10 years of tenure, the OR of the group working more than 40 hours and up to 52 hours was 1.27 (95% CI, 0.84-1.93), 1.05 (95% CI, 0.62-1.76) for the group working more than 52 hours and up to 60 hours, and 1.37 (95% CI, 0.71-2.65) for the group working more than 60 hours.

## Discussion

In this study, the results of the analysis on weekly working hours of full-time wage workers among the 11th KLIPS study participants showed that the average number of weekly working hours was 48.8 hours, with the male workers showing a higher rate of working hours of more than 52 hours per week than female workers. It has been found that 30.6% of the participants of this study worked for more than 52 hours per week, which is the permitted standard of the Labor Standards Act. This is similar to the results of the 4th Korea National Health and Nutrition Examination Survey conducted on full-time wage workers, in which the weekly working hours were 48.3 hours and the rate of employees working more than 52 hours per week was 29.6% [19]. Considering both sources of data, around 30% of Korea’s full-time wage workers are working for more than the number of hours permitted by the Labor Standards Act, and the reason for this is assumed to be the fact that Korea’s Labor Standards Act does not have a separate regulation for working on Sundays and holidays. Moreover, the average number of working hours per week of the study participants, which was 48.8 hours, was 11.3 hours more than the average weekly working hours of European countries found in the 5th European Working Conditions Survey, which was 37.5 hours [11]. Korea’s actual conditions of long working hours can thus be seen not only through the OECD reports but also through this study.

The results of this study on the relationship between weekly working hours and the self-rated health level showed that the health level was getting worse in a dose-dependent manner as the number of working hours increased, and the group working more than 60 hours per week showed the worst health level. Such an effect was considerably more significant in the case of female workers and workers with more than one year of tenure. Thus far, studies using domestic Korean data on the health effects of long working hours have mostly been related to mental health. It has been reported that in a study conducted on public sector workers, the size of the group showing high-risk depressive symptoms increased with an increase in the number of weekly working hours and the OR of depressive symptoms in workers working more than 10 hours daily more than seven times a month was 1.63 (95% CI, 1.10-2.43) [18]. The results of the 4th Korea National Health and Nutrition Examination Survey conducted on full-time wage workers revealed that the OR of depressive symptomatology for the group working more than 60 hours per week was 1.62 (95% CI, 1.20-2.18) as compared to the group working less than 52 hours per week [19]. Another study conducted on full-time wage workers of the 4th Korea National Health and Nutrition Examination Survey reported that the OR of suicidal ideation for the group working more than 60 hours per week was 1.38 (p = 0.020) as compared to the group working more than 40 hours and up to 51 hours per week [17]. This study found that long working hours have negative impacts not only on workers’ mental health but also on their self-rated health level, showing an increase in fair, poor, and very poor self-rated health with an increase in the number of working hours; further, this unhealthy rate was the highest when the number of working hours exceeded 60 hours per week, which is the standard of overwork.

In previous studies, the mechanism of the impact of long working hours on health was explained by dividing it into the direct pathway of a physiological recovery mechanism and the indirect pathway of a behavioral lifestyle mechanism [23-26]. The physiological recovery mechanism explained that insufficiency in recovery is caused by a lack of sleeping hours resulting from long working hours and that this has a negative impact on workers’ health [27-31]. Further, it has been reported that insufficient recovery from labor can cause blood pressure elevation [32], sympathetic nerve activation [1,24], and an increase in the morbidity rate of cardiovascular diseases [33]. The behavioral lifestyle mechanism explains that changes in lifestyle such as an increase in the percentage of smoking and alcohol-consuming groups and decrease in the percentage of workers exercising regularly due to long working hours have a negative impact on the workers’ health [34-36]. It also explains that there is an increase in the number of workers showing unhealthy eating habits including an increase in the intake of foods high in fat or calories and decrease in the consumption of foods beneficial to health such as vegetables and fruits [37,38]. Although the physiological recovery mechanism could not be verified through this study, the health impact of long working hours could be found to reach a certain level through the behavioral lifestyle mechanism. The results of this study showed that the percentage of the group working more than 52 hours was higher in the smoking, alcohol-consuming, and non-exercising groups than in the non-smoking, non-alcohol-consuming, and non-exercising groups in the cases of both male and female workers (Tables 2 and 3).

In this study, the behavioral lifestyle mechanism was identified through the fact that the smoking, alcohol-consuming, and non-exercising groups with unhealthy lifestyles had a higher percentage of workers working long hours and the self-rated health level in accordance with health-related behavior characteristics showed a higher percentage of unhealthy workers among the smoking, non-alcohol-consuming, and non-exercising groups in the cases of both male and female workers. The psychological impact of alcohol-consumption, specifically stress reduction, may be explained for the high percentage of unhealthy workers in the non-alcohol-consuming group. In a social survey conducted in Korea in 2012, the results showed that proportion of respondents who answered that they were in good health in a self-health evaluation was 45.6% for the alcohol-consuming group and 34% for the non-alcohol-consuming group [39]. This finding may be explained by a psychological effect, in that the alcohol-consuming group may believe they are in better health than they are. The positive effects of mild to moderate alcohol-consumption can also be considered. In a previous study on the risk of alcohol-consumption and cardiovascular disorders, it was proven that irrespective of the type of alcohol, mild to moderate alcohol-consumption may lower the risk of coronary artery disease [40]. Another study reported that a moderate intake of wine decreases oxidative stress and reduces atherosclerosis by enhancing cholesterol efflux from the vessel walls and inhibiting platelet adhesion [41].

In this study, the result of reanalysis after gender stratification showed a more noticeable tendency of having a poorer self-rated health level as the number of working hours increased in the case of female workers. This is mainly attributed to the burden of household chores for females. According to the Time Use Survey conducted in 2009 in Korea, the house-work time for weekdays was 27 min for husbands and 185 min for wives in a two-income household [42]. Therefore, female workers use most of the remaining time after work on household chores, leaving insufficient time for recovery and being more vulnerable to poor health due to long working hours. Korea’s gender difference in the labor market should also be considered. Korea’s labor market has a gender separation structure with male workers mostly taking up the higher level of occupational status with high wages and high job control and the female workers taking up the lower level with low wages and low job control; it has been reported that the US labor market also shows a similar pattern [22]. This is mainly attributed to the fact that females, excluding some women with professional jobs, experience career discontinuity due to pregnancy and childbirth and show a tendency to take jobs unrelated to their past career during the reemployment process. With respect to the distribution of the age structure of this study, the percentage of the male subjects aged 35 to 44 years was the highest, at 32.8%, but the percentage of female subjects aged 25 to 34 years was the highest, at 37.6%. According to the job strain model presented by Karasek et al., the group with high job control was classified as the active group, while the group with low job control was classified as the high job strain group, in the case of high work demands [43]. It has been identified that the group with a high job strain level will be affected with negative health impacts such as having a relatively higher risk of diabetes or cardiovascular diseases as compared to the other groups [44,45]. Thus, there is a possibility that female workers working long hours may show a relatively high job strain level due to career discontinuity, which leads to the belief that female workers are more vulnerable to poor health when working long hours. However, there is a need for additional studies in the future as this study does not cover psychosocial stress.

In this study, the self-rated health level was reanalyzed after tenure stratification. As a result of this, the OR of a poor self-rated health level increased with a dose–response pattern related to long working hours when the tenure exceeded one year and the effect was attenuated when the tenure exceeded 10 years. Further, it was suggested that the cumulative exposure time until the working hours affect workers’ health be estimated. This is because for the physiological recovery mechanism, a certain amount of cumulative exposure time is required in order for the insufficient recovery time resulting from working long hours to have a negative impact on the workers’ health and for the behavioral lifestyle mechanism, a certain amount of exposure time is required for workers exposed to long working hours to make changes in lifestyle and for this to have a negative impact on the workers’ health. The results of a systematic review analyzing the effect of the lifestyle changes on the metabolic syndrome showed that the group that changed to a healthy lifestyle showed a noticeable recovery rate from the metabolic syndrome that was two times higher than that of the comparison group after an average of one year had passed [46]. In another study, the group that maintained the changed lifestyle for more than one year was reported showing significant improvements in the risk factors of cardiovascular diseases such as obesity or blood lipid disorders as compared to the comparison group [47]. In this study, a significant difference in the self-rated health level was observed only in workers with a tenure of more than one year, which means that a cumulative exposure time of more than one year is expected in order for long working hours to have a negative impact on workers’ health. Further, the health effects brought about by an improved lifestyle can be believed to be the reason why the OR of the poor self-rated health level caused by the high number of working hours decreased for the group with a tenure of exceeding 10 years. The healthy worker effect is a bias observed commonly in the field of occupational epidemiology that arises because those with health problems tend to drop out of the working population, leaving the working population relatively healthier than the general population [48]. There is a possibility that only relatively healthy workers work in the same workplace for more than 10 years because those with health disorders resulting from a negative health impact of long working hours would have stopped working in the middle of the career. Therefore, workers working for a long period of time of more than 10 years would end up being relatively healthy people with a low percentage of selecting “unhealthy” in the self-rated health level; this implies, that the data collected on the impact of working hours on the self-rated health level may be distorted, hiding the true extent of the impact of long working hours.

This study has the following limitations: First, in this cross-sectional study, there is a limitation in explaining the relationship between the number of working hours and the self-rated health level by analyzing just the 11th year (2008) data among the longitudinal KLIPS data. Second, there is a possibility of an information bias because the KLIPS data were collected using self-report through questionnaires. Third, the research results cannot be said to be fully adjusted because there is insufficient data to verify the study participants’ alcohol consumption, past medical history, past work experience and number of working hours. Despite these limitations, this study identified the relationship between the number of working hours and the self-rated health level by using the KLIPS data, which represent Korea’s urban household members. The results of this study showed that after controlling for various relevant variables, the group working more than 60 hours per week exhibited a significantly increased risk of poor self-rated health as compared to the group working less than 40 hours per week and that such an impact was much more significant in the case of the female workers and the workers with more than one year of tenure. Long-term follow-up studies may be needed to show the various health impacts of long working hours.

## Competing interests

Authors declare that they have no competing interests.

## Authors’ contributions

J-TS designed this study and made a draft of this manuscript. GL, JK and J-WP were analyzed the data. HC did technical support. SL did critical revision of the manuscript. All authors read and approved the final manuscript.
